# The Influence of Patient-Centered Communication on Children’s Anxiety and Use of Anesthesia for MR

**DOI:** 10.3390/ijerph20010414

**Published:** 2022-12-27

**Authors:** M. Conceição Castro, Isabel Ramos, Irene Palmares Carvalho

**Affiliations:** 1 Department of Radiology, Centro Hospitalar Universitário de São João–Alameda Professor Hernâni Monteiro, 4200-319 Porto, Portugal; 2 Faculty of Medicine, University of Porto-Alameda Professor Hernâni Monteiro, 4200-319 Porto, Portugal; 3 Department of Clinical Neurosciences and Mental Health, Faculty of Medicine, University of Porto-Alameda Professor Hernâni Monteiro, 4200-319 Porto, Portugal; 4 CINTESIS@RISE, Faculty of Medicine, University of Porto-Alameda Professor Hernâni Monteiro, 4200-319 Porto, Portugal

**Keywords:** anesthesia, children, magnetic resonance imaging, patient-centered communication, toy simulation

## Abstract

Background: The aim of this study was to inspect the influence of patient-centered communication (PCC) with 4- to 10-year-old children on the use of anesthesia for magnetic resonance imaging exams (MRs). Methods: A total of thirty children received the PCC and pre-simulated the exam with an MR toy. Another 30 children received routine information about the MR and pre-simulated the exam with the toy. Anesthesia use in these two groups was additionally compared with a previously existing group of children (*n* = 30) who had received only routine information about the exam (CG). Children’s anxiety was assessed with a self-report question plus heartbeat frequency. Children’s satisfaction was assessed through several questions. The analyses were based on group comparisons and regression. Results: A total of two children (7%) in the PCC + simulation group used sedation compared with 14 (47%) in the simulation group and 21 (70%) in the CG. Differences between the PCC + simulation and the other two groups were significant (*p* < 0.001), although not between the simulation and the CG. The decrease in anxiety was significantly greater (self-reported *p* < 0.001; heart rate *p* < 0.05) and satisfaction was higher (*p* = 0.001) in the PCC + simulation, when compared with the simulation group. Reduced anxiety was associated with less anesthesia use (OR 1.39; CI 1.07–1.79; *p* = 0.013). Conclusions: PCC + simulation was more effective than simulation and routine practice in decreasing children’s anxiety, increasing satisfaction, and reducing the use of anesthesia for MRs.

## 1. Introduction

Magnetic resonance imaging (MR) scans are non-invasive, painless exams that provide high-quality images of almost all regions of the body without using ionizing radiation [1]. Despite these advantages, MR scans can be distressing for children, and previous studies have shown that more than two thirds experience this exam with feelings of moderate to severe anxiety [2,3]. This is of particular importance for health institutions because a considerable number of children undergo MR exams only when anesthesia is used. The use of anesthesia for MRs is logistically challenging, time and resource consuming [4,5], and entails risks of adverse health effects, including respiratory depression, airway obstruction, bronchospasm or laryngospasm, aspiration pneumonia, cardiovascular depression, arrhythmia, hypotension, and adverse reactions to the drugs that are used such as anaphylaxis [6,7,8]. Although under ideal conditions severe complications are extremely rare, the most feared risk is the inability of patients to maintain their airway functioning due to a decreased level of consciousness [9,10]. In addition, some studies have shown an association between anesthesia exposure and neurodevelopmental difficulties [11,12,13,14].

Given these disadvantages, different strategies have been explored and have shown success in reducing anxiety and the need for sedation among children who undergo MR exams, including animal-assisted therapy, the use of audiovisual systems, behavioral desensitization programs, among others [3,15,16,17,18,19,20,21,22,23,24]. For example, the use of animal-assisted therapy as a non-pharmacological intervention involving the child sitting next to a dog, petting it, and playing with it under the coach’s supervision reduced anxiety and prevented the use of sedation for MR exams [17]. The use of clowns in addition to live music and dog interactions also has yielded encouraging results, reducing anxiety and the need for sedation among children aged 4 to 11 years old who underwent MR exams [20]. The installation of audiovisual systems as an alternative to child sedation for MRs has been successful as well [3,18]. Practicing with mock MRs by simulating an exam before the actual diagnostic resonance promotes familiarization with the equipment, sounds, and procedures, namely through play, and works as a behavioral desensitization strategy that has been effective in reducing the rate of anesthesia used for MRs when compared to the standard practice [21,22,23].

The effectiveness of such strategies is often premised on a child-centered approach that helps to create a non-threatening, attractive, and meaningful universe that is adapted to the child. However, despite the long-standing recognition of the importance of child-centered approaches [25,26,27,28,29,30,31,32,33], the role of a patient-centered model of communication (PCC) with the child about the MR exam in the preparatory clinical encounter is unclear, particularly when compared with other child-centered strategies that are also employed to reduce child anxiety and anesthesia use. Patient-centered approaches have been effective in reducing anxiety and improving clinical outcomes and satisfaction with care among adults in various clinical contexts when compared with the routine provision of information [34,35,36,37]. It is possible that the effectiveness of a patient-centered model of communication is superior when applied to children as well. To the best of our knowledge, research has not examined the effect of a PCC on the use of anesthesia for MRs among children.

To bridge this gap, the goal of the current study was to inspect the influence of a PCC on children’s use of anesthesia for MR exams, comparing that with other child-friendly forms of providing information in the preparatory encounter. If the PCC is superior, then a reduction in child’s anxiety and increased satisfaction with care, translating into decreased use of anesthesia for the MR exam, should be greater for the PCC than for other child-centered, rapport-building models of communication. The PCC model is presented in Box 1.

Box 1 **Patient-centered communication (PCC).**

A patient-centered approach to the clinical encounter entails consideration of the patient’s individual needs and is premised on the purposeful elicitation of the patient’s own perspective about the problem [38,39,40]. Based on the notion of effective communication skills that are used for these purposes (e.g., starting with open questions, listening attentively without interrupting, reflecting, paraphrasing, using appropriate non-verbal communication), this model contrasts sharply with (more directive) routine procedures of providing information and then checking for doubts, which might leave out important issues that the patient lacked the opportunity to share, or introduce (excessive) information that the patient was not interested in obtaining [41,42].

When applied to children [43], the PCC is thus premised on a dynamic dialogue with the child in the preparatory clinical encounter. It entails purposefully eliciting the child’s ideas, expectations, fears, concerns, or questions regarding the MR exam, first (through effective communication skills), and then providing responses (including empathic responses) that are tailored at the expressed needs of the child.

Unlike in routine, more directive (professional-centered) clinical practice, the PCC focuses on the patient, not on the information to be provided. The point of departure for the interaction is thus the perspective of the child, whose lead the professional follows rather than focusing on the procedures, forms, or protocols.

## 2. Materials and Methods

### 2.1. Participants

The study’s population consisted of all children scheduled for exams in the MR unit of the Radiology Service at one of the largest general hospitals in Portugal between January and July of 2019 and of 2020. The inclusion criteria were (i) ages between four and 10 years old and (ii) a medical request for an MR in the hospital. The exclusion criteria were (i) prior MR experiences; (ii) presence of cognitive, psychiatric, or neurological conditions that prevented the understanding of communication; and (iii) the presence of intra-body devices that impeded performance of the MR exam. This and other information were obtained from the children’s medical records (stored in the Siima information system where the institution’s diagnostic exams are managed) and confirmed through a socio-demographic and clinical questionnaire that was applied to the participants for the purposes of this study.

**Figure 1:**
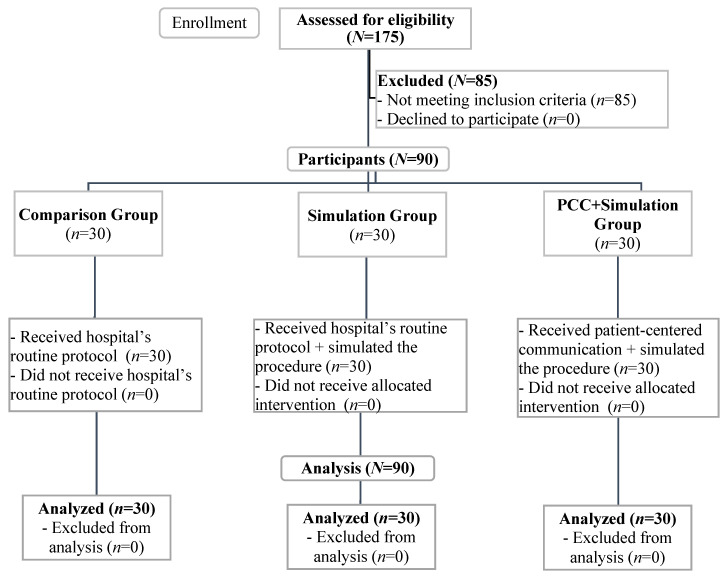
Flow diagram of enrollment, eligibility screening, and analysis.

Of all 175 initially eligible children, 90 (51.4%) were included in the study. The exclusion of 85 children had to do mostly with the fact that they had undergone MR exams previously (*n* = 65 children), followed by the presence of a condition that prevented the understanding of communication (*n* = 20 children). Figure 1 depicts the study’s enrollment flow chart.

### 2.2. Measures

In addition to the socio-demographic and clinical information that was mentioned above, the measures of child anxiety were used before and after the child–professional interaction, and the measures of satisfaction were used after the child–professional interaction. All were applied before the MR scans.

Children’s anxiety was assessed through a self-report question and through objective heart rate frequency. The question asked children, “How do you feel right now?” and was answered on a 5-point Likert-type scale ranging from 1—“very calm” to 5—“very scared”. This scale was presented as a facial image scale (FIS) which is ideal for children who do not yet know how to read. The FIS is a valid instrument above three years of age in the clinical context [44] that provides the child’s direct perception, thus preventing the threats to validity that are associated with the use of standardized questionnaires at young ages. The word options that were associated to the faces were validated independently by three psychologists with expertise in the area of pediatrics after the revision of a list of possible words. The validation was consensual regarding the simplest, most accessible words for the children. An open circle was added below each face for children to paint their choices (cross-validated by the investigator against the faces/words of their choice), thus encouraging their active participation for a greater involvement in the situation [45].

The physiological measure of the child’s state of anxiety, i.e., heart rate frequency, was obtained with a pulse oximeter that was connected to the child’s finger. This small, wireless, portable device was considered as a non-invasive tool that provides a quick and precise measurement.

The child’s satisfaction with the interaction was assessed through a series of six questions. The first, 1—“How was your experience today?” was responded on a 5-point Likert-type scale ranging from 1—“I hated it” to 5—“I loved it” on the FIS, according to the procedures that were described above. The remaining questions asked for a “yes”, “no”, or “I don’t know” response to whether this experience: 2—“Helped me feel calmer”; 3—“Decreased my fears”; 4—“Taught me not to move”; 5—“Taught me to tolerate loud noises”; and 6—“The next time you come to the hospital, would you like to repeat this experience?” The reminder, “Here there are no right or wrong answers, what matters is what you feel” accompanied all these procedures.

The measure of anesthesia use was the number of children who needed anesthesia for the MR exams. This information was obtained from the child’s medical records.

### 2.3. Procedure

All eligible children and their legal representatives were invited to participate in the study when they went to the hospital for their MRs. All parents (or children’s legal representatives) accepted to participate (100%) and signed an informed consent form. The study was conducted according to the guidelines of the Declaration of Helsinki and approved by the Ethics Committee for Health of São João University Hospital Center/Faculty of Medicine of University of Porto (No. 333/19).

Children that were scheduled for their MRs between January and March of 2020 formed the experimental group 1 (EG1). Children that were scheduled between April and July of 2020 formed the experimental group 2 (EG2). Although these two groups constituted the study, it seemed interesting to additionally compare the use of anesthesia with a third, pre-existing group of children who had attended the unit throughout the same period of time in the previous year (CG).

**Figure 2:**
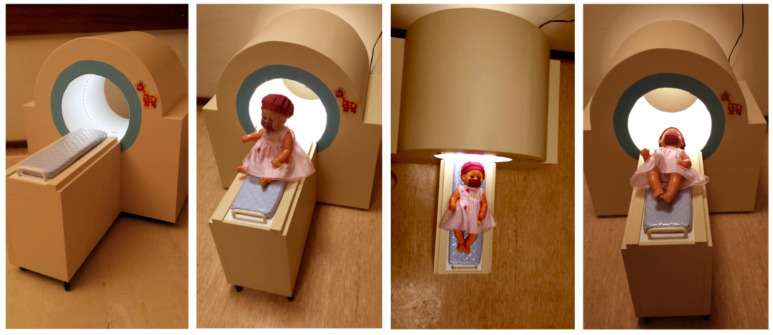
Mini-resonance toy imitating the looks and sounds of a real MR, built for this study.

The CG received only the usual routine protocol in the MR unit, consisting of general information about the procedures of the MR exam. In EG1, the children received this general information protocol about the exam and simulated the procedure with a “mini-resonance” toy that imitates the looks and sounds of a real MR (“Sim” group) that was built for this study (Figure 2). The intervention in EG2 included simulation with the mini-resonance toy and the patient-centered communication (“PCC + Sim” group), consisting of following the child’s leads (Box 1).

The procedures were otherwise the same in all three groups. Following the institution’s routine, the information was conveyed in a child-friendly manner in the three groups. The duration of the interaction (since arrival until entering the MR exam room) was the institution’s standard duration for all groups (about 30 min). Preparing the child for the MRs took approximately 15 min in each group. This preparation was conducted by an experienced radiology technologist with expertise in communication skills based on the Calgary Cambridge Guide [41].

To ensure the consistent implementation of each type of intervention in the Sim and in the PCC + Sim groups, respectively, without risking contamination, a sequential block design was chosen over a randomized design so that one type of intervention was fully complete with one group of children before the other began. In addition, monitoring of the correct application of each type of intervention occurred constantly through frequent meetings between the researchers. When the restrictions that were associated with the COVID-19 pandemic began in March of 2020, the Pediatric Radiology Unit functioned exactly as before through the reduction of the number of children that were received per day.

Before the intervention (T0), the child’s anxiety was assessed in the two study groups (Sim and PCC + Sim) through the self-report question and registration of heart rate frequency (measured after a resting period of 10 min). After the intervention (T1), children in the Sim and PCC + Sim groups were assessed regarding their anxiety again and regarding their satisfaction with the interaction. Both moments of assessment took place before the MR exams and were conducted by the same radiology technologist with expertise in communication skills, who ensured the exact same procedures across the groups. A pediatric cardiologist independently supervised the procedures and validated all data collection. A different team (blind with regards to the group to which the child was assigned) performed the MR exams as usual. All children underwent either general radiology (body, musculoskeletal) MRs targeting the body, or neuroradiology (brain, spine) MRs, targeting brain problems. Neuroradiology MRs lasted 10 to 15 min longer, on average, than did general radiology MRs.

### 2.4. Analyses

Variables were expressed as the mean and standard deviation (for continuous variables), median, and interquartile range (for ordinal variables) and frequency and percentage (for categorical variables). For numerical variables where *n* = 30, the sample was considered to follow a normal distribution according to the Central Limit Theorem. The homogeneity of variances was confirmed through Levene’s test. Thus, the *t*-test for independent samples was applied to compare the means of two groups and an ANOVA was used for more than two groups. Paired-samples groups were analyzed with the *t*-test for paired samples. Regarding the ordinal variables, for both anxiety and satisfaction scales, the Mann–Whitney test was used to compare two groups of independent samples and the Wilcoxon test was applied to compare paired samples. For categorical variables, the frequencies of two independent groups with two or more categories were compared through the Chi-square test. Logistic regression was conducted to inspect the role of changes in anxiety on anesthesia use. All statistical analyses were performed in IBM SPSS version 26. The level of significance was set at 5%.

## 3. Results

Table 1 presents the sample’s characteristics of the 90 participants. The mean age was 6.5 years old and the proportion of boys and girls was the same (50%). Half of the children underwent neuroradiology exams and the other half received general radiology MRs. Most were referred from outpatient care (76.7%).

Each study group comprised 30 children. The three groups registered non-significant differences at baseline regarding age (*p* = 0.343), gender (*p* = 0.301), type of exam (*p* = 0.202), and type of referral (*p* = 0.271) (Table 1).

### 3.1. Anxiety

Before the intervention (T0), the children’s anxiety levels in the simulation and PCC + simulation groups were not significantly different (*p* = 0.288). After the intervention (T1), the levels of anxiety decreased significantly in both groups (simulation: *z* = −2.553; *p* < 0.05 and PCC + simulation: *z* = −4.275; *p* < 0.001). However, they were significantly lower in the PCC + simulation when compared with the simulation group, *U* (*N* = 60) = 138; *z* = −4.782; *p* < 0.001 (Table 2).

The children’s heart rates followed a similar pattern. The values in the simulation and PCC + simulation groups were not statistically different before the intervention (T0) (*p* = 0.291). After the intervention (T1), the children’s heart rates decreased in both groups (simulation: *t*(29) = 2.747; *p* < 0.05 and PCC + simulation: *t*(29) = 9.011; *p* < 0.001) but were significantly lower in the PCC + simulation when compared with the simulation group, *t*(46) = 2.966; *p* < 0.05 (Table 2).

### 3.2. Satisfaction

Satisfaction with the interaction was significantly greater in the PCC + Sim group for all aspects asked when compared with the Sim group (Table 3). Satisfaction with the interaction (item 1) received the maximum level on the scale (5- “I loved it”) from the children in the PCC + Sim group (*U* (*N* = 60) = 243: *z* = −3.357; *p* < 0.05), unlike in the Sim group (median = 4). Accordingly, almost all children in the PCC + Sim group reported that they 6- “would like to repeat the experience in a future visit to the hospital”, whereas similar proportions of children in the Sim group responded affirmatively and negatively to this question (Table 3).

Regarding the remaining items (Table 3), more children in the PCC + Sim group (100%) than in the Sim group (66.7% and less) reported that the interaction 2—“helped them feel calmer” (χ^2^ (1, *N* = 60) = 12; *p* < 0.05), 3—“less afraid” of the exam (χ^2^ (1, *N* = 60) = 12; *p* < 0.05), and 4—“taught them not to move” (χ^2^ (1, *N* = 60) = 23.721; *p* < 0.05). In addition, whereas 93.3% of the children in the PCC + Sim group reported that the experience 5—“taught them to tolerate loud noises”, 70.0% of children in the Sim group answered the opposite.

### 3.3. Use of Anesthesia

Considering the whole sample together, the proportion of children using anesthesia was not significantly different with regards to gender (17 boys, or 38%, and 20 girls, or 44%), χ^2^ (1, *N* = 90) = 0.413; *p* = 0.520, and referral origin (27 cases, or 39%, from outpatient care and 10 cases, or 48%, from inpatient care), χ^2^ (1, *N* = 90) = 0.479; *p* = 0.489. However, children who used anesthesia were significantly younger (*M* = 5.54; *SD* = 1.70) than were children who did not use it (*M* = 7.23; *SD* = 1.80), *t* (88) = −4.424; *p* < 0.001. Children also used anesthesia significantly more often for neuroradiology (27 children, or 60%) than for general radiology MRs (10 children, or 22%), χ^2^ (1, *N* = 90) = 13.264; *p* < 0.001.

The two study groups and the additional comparison group were not significantly different with regards to all these characteristics, as mentioned earlier. However, the number of children who used sedation for their MR exams was 21 in the CG (corresponding to 70% of this group), 14 in the Sim group (or nearly half of this group, corresponding to 47%), and 2 in the PCC + Sim group (or 7%). The difference between the CG and the Sim group was statistically non-significant (the statistical power of this test for a small effect size of 0.2 was 0.47). In contrast, the PCC + Sim group differed significantly from both the CG, χ^2^ (1, *N* = 60) = 25.452; *p* < 0.001, and the Sim group, χ^2^ (1, *N* = 60) = 12.273; *p* < 0.001. An additional regression analysis showed that the reduction in anxiety (assessed through heartbeat frequency) predicted (less) sedation use across both the Sim and the PCC + Sim groups (OR 1.39; CI 1.07–1.79; *p* = 0.013), adjusting for other factors (Table 4).

## 4. Discussion

This study’s results show that a dialogue-based PCC with the child at the time of the preparatory clinical encounter is a viable and effective alternative to the use of anesthesia among 4- to 10-year-old children who need a diagnostic MR. The PCC intervention with pre-simulation of the procedures was associated with significantly decreased use of anesthesia for the MR examinations when compared to the routine practice of providing information along with pre-simulating the procedures (simulation group) and to the routine practice of providing information alone (CG). The fact that there was no significant difference between the Sim group and the CG was surprising in light of previous research indicating that behavioral desensitization through simulation is more effective in reducing anesthesia use for MR exams than simple routine practice [21,22]. However, the observed statistical power of this test was small and a significant effect might be visible with a larger sample of participants. Nevertheless, the results pointed unequivocally to the greatest effectiveness of children’s preparation when the PCC was used.

Similar to previous research using the FIS [17], children’s anxiety levels were initially high. The reduced use of anesthesia in the PCC + Sim group occurred following a greater decrease in anxiety and greater satisfaction with the interaction in this PCC + Sim group when compared to the simulation group. The objective measure of anxiety (heart rate frequency) corroborated the children’s self-reported decrease in anxiety after the intervention, yielding equivalent results, and the regression analysis added evidence in support of the role of anxiety reduction on the reduction of anesthesia use.

This study thus extends the positive effects of a patient-centered model of communication [34,35,39] to the context of MRs with pediatric populations, providing support to the importance of actively encouraging even young children to express their own ideas and feelings about these exams and of providing responses and emotional support that is tailored to each child’s individual needs. The comparatively lesser success of the simulation model (and of the routine-information protocol) in this study suggests that, without the PCC, there is a risk of centering the interaction, and the information provided, on the professionals’, parents’, or procedures’ (not the patient’s) needs, at least in part, which will fail to fully reassure the child [31,32,34,39] even when child-centered, rapport-building strategies are used. An implication is that strategies that are employed with children in this context might be more effective if accompanied by a PCC whenever possible.

The fact that, when all the children in the three groups were considered together as a whole, anesthesia was used significantly more often for MRs with younger, rather than older children, might have to do with greater anxiety and difficulty in remaining still during the exam at younger ages [2,3]. Anesthesia was used also significantly more often in neuroradiology than in general radiology MRs perhaps because of children’s brain-associated difficulties involved in neuroradiology exams that might interfere with their behavior during the scan, or due to the longer time that these exams take on average compared to general radiology MRs, which might add to children’s difficulties in remaining immobile throughout the entire exam. Neuroradiology MRs also involve head coils, which might contribute to the sensation of having the head covered and increase the child’s discomfort. Nevertheless, both age and type of (neuroradiology or general radiology) exam were evenly distributed across groups and the results showed that, even in these challenging situations, anesthesia was largely unnecessary when the preparation of the child for the exam was based on the PCC.

Although restrictions that were associated with the COVID-19 pandemic began in March of 2020, there were no reasons to believe that these circumstances influenced the results, given the hospital’s decision to maintain pediatric MR exams as unchanged and functioning as before. Still, school closing and social distancing might affect children’s susceptibility to communication, although there was no evidence of such changes in the context of this study (for example, comparing the interaction dynamics of the children in the PCC + Sim group with the pre-COVID-19 Sim group), which was restricted to a formal clinical encounter about MRs with professional adults in a health setting.

### Strengths and Limitations

The study’s exclusion criteria led to the elimination of nearly half of the initially eligible population of children, limiting the sample size. However, inclusion of these participants in the study to increase the sample size could have biased the results. Children with previous MR experiences might undergo these exams either more easily (due to familiarization with the procedures) or less easily (due to previous negative experiences with MRs) than would children without such experiences. Also, group assignment was not randomized. This decision was made to ensure the rigorous application of each intervention strategy without risking any possible contamination between them. Based on past clinical experience with MRs at the hospital, systematic differences among the groups of children were not anticipated and, despite a lack of randomization, the groups registered non-significant differences at baseline regarding the various characteristics that were studied. Finally, data on anxiety and satisfaction were unavailable for the additional comparison group (CG), limiting the analyses of this group to anesthesia use. The PCC is based on direct dialogue with the child and is not applicable to children who are unable to speak or to understand language, but proved effective even at young ages.

Despite these limitations, the rigorous application of the experimental design for causal inferences to the two intervention groups, and the choice of measures that are best suited for young children in the clinical setting, including the combination of self-report and physiological, quantitative measures, maximize the validity of the results and the robustness of the conclusions. Future studies could focus on the effects of preparing the child by using this PCC without the simulation component or, alternatively, with other toys and media that help to build rapport with the child for inspection of whether the superior results that were obtained with this communication strategy remain. The PCC might apply to other pediatric areas as well, such as orthopantomography (i.e., imaging tests that are fundamental in dentistry), justifying future studies.

## 5. Conclusions

This study’s results showed the positive effects of patient-centered communication on children’s anxiety, satisfaction, and reduced use of sedation for MR exams at ages 4- through to 10-years old. The PCC is based on direct dialogue with the child and proved effective even at young ages. These results underline the importance of this type of PCC intervention in clinical contexts. Professionals’ training on this patient-centered communication model is warranted, along with the institutional endorsement of this model of interaction for inclusion of such interventions in the routine preparation of children for imaging exams.

## Figures and Tables

**Table 1:** Sample characteristics.

		Total	CG	Simulation	PCC + Simulation	Group Difference
		*N* = 90	*n* = 30	*n* = 30	*n* = 30	*p*-Value
Age (years)-*Mean* (*SD*)		6.53 (1.95)	6.47 (2.05)	6.20 (2.09)	6.93 (1.70)	0.343 ^a^
Gender-*n* (*%*)	Boys	45 (50.0%)	18 (60.0)	12 (40.0)	15 (50.0)	0.301 ^b^
	Girls	45 (50.0%)	12 (40.0)	18 (60.0)	15 (50.0)	
Exam type-*n* (*%*)	Neuroradiology	45 (50.0%)	19 (63.3)	13 (43.3)	13 (43.3)	0.202 ^b^
	General radiology	45 (50.0%)	11 (36.7)	17 (56.7)	17 (56.7)	
Referral-*n* (*%*)	Outpatient care	69 (76.7%)	21 (70.0)	22 (73.3)	26 (86.7)	0.271 ^b^
	Inpatient care	21 (23.3%)	9 (30.0)	8 (26.7)	4 (13.3)	

SD—Standard deviation; CG—Comparison group; ^a^ One-way ANOVA; ^b^ Chi-square test.

**Table 2:** Children’s anxiety levels before and after the intervention in the simulation group (without the PCC model) and in the PCC + simulation group.

Anxiety	Group	T0	T1	*p* [T1-T0]
Self-reported-*Median* (*IQR*) ^1^	“Sim”	4 (2–4)	3 (2–4)	0.011 ^a^
	“PCC + Sim”	3 (2–4)	2 (1–2)	<0.001 ^a^
	*p* [“Sim”-“PCC + Sim”]	0.288 ^b^	<0.001 ^b^	
Heart rate (bpm)-*Mean* (*SD*) ^2^	“Sim”	96.27 (15.39)	91.60 (16.66)	0.010 ^c^
	“PCC + Sim”	92.37 (12.86)	81.20 (9.56)	<0.001 ^c^
	*p* [“Sim”-“PCC + Sim”]	0.291 ^d^	0.005 ^d^	

^1^ IQR—interquartile range; ^2^ *SD*—standard deviation; ^a^ Wilcoxon test; ^b^ Mann–Whitney test; ^c^ *t*-test for paired samples; ^d^ *t*-test for independent samples; bpm-beats per minute; T0-evaluation before the intervention; T1-evaluation after the intervention.

**Table 3:** Children’s satisfaction with the interaction in the simulation group (without the PCC model) and in the PCC + Sim group.

Satisfaction with the Interaction		“Sim”	“PCC + Sim”	*p* [“Sim”-“PCC + Sim”]
1—“Loved it”-*Median* (*IQR*) ^1^		4 (3–5)	5 (4–5)	0.001 ^a^
2—“Helped me feel calmer”-*n* (*%*)	Yes	20 (66.7)	30 (100.0)	0.001 ^b^
	No	10 (33.3)	0 (0.0)	
	I don’t know	0 (0.0)	0 (0.0)	
3—“Decreased my fears”-*n* (*%*)	Yes	20 (66.7)	30 (100.0)	0.001 ^b^
	No	10 (33.3)	0 (0.0)	
	I don’t know	0 (0.0)	0 (0.0)	
4—“Taught me not to move”-*n* (*%*)	Yes	13 (43.3)	30 (100.0)	<0.001 ^b^
	No	17 (56.7)	0 (0.0)	
	I don’t know	0 (0.0)	0 (0.0)	
5—“Taught me to tolerate loud noises”-*n* (*%*)	Yes	9 (30.0)	28 (93.3)	NA
	No	21 (70.0)	1 (3.3)	
	I don’t know	0 (0.0)	1 (3.3)	
6—“Would like to repeat it next time”-*n* (*%*)	Yes	14 (46.7)	28 (93.3)	NA
	No	12 (40.0)	1 (3.3)	
	I don’t know	4 (13.3)	1 (3.3)	

^1^ IQR—interquartile range; ^a^ Mann–Whitney test; ^b^ Chi-square test; NA = not applicable because the assumptions of the Chi-square test were not met.

**Table 4:** The odds of (less) anesthesia use for reduced anxiety across groups (“Sim” and “PCC + Sim”), adjusting for other possible factors (*N* = 60).

	(Less) Anesthesia Use	
	OR (95% CI)	*p*
Reduced anxiety (heartbeat frequency)	1.39 (1.07–1.79)	0.013
Age	3.26 (1.43–7.43)	0.005
Male gender	1.51 (0.23–10.15)	0.671
General radiology exams	10.69 (1.20–94.81)	0.033
Referral from inpatient care	4.07 (0.32–51.37)	0.278

(Less) anesthesia use = 1—not used (*vs*. 0—used). OR = odds ratio. CI = confidence interval. Reduced anxiety calculated by subtracting heartbeat values at T1 from the values at T0.

## Data Availability

This clinical trial can be found at the ClinicalTrials.gov public web site (NCT05165576).
